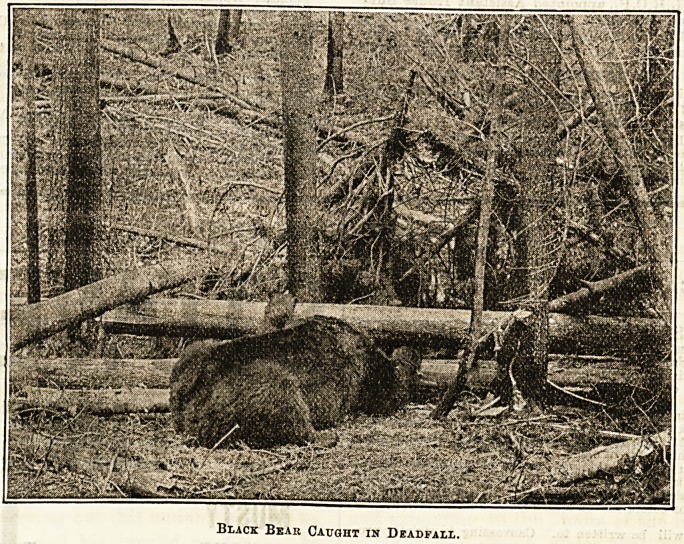# The Book World of Medicine and Science

**Published:** 1894-03-17

**Authors:** 


### THE BOOK WORLD OF MEDICINE AND SCIENCE.

### BOOK REVIEWS.

LIFE IN THE BACKWOODS. From original Photographs taken by J. TURNER-TURNER. (The London Stereoscopic and Photographic Company, Limited.)

How do you live in the backwoods, and how do you keep warm with the thermometer between 30 and 40 below zero? Mr. Turner-Turner supplies the response to these questions, asked of those who wander, as he has done, among the rockies in search of big game, in a series of wonderfully fascinating pictures which tell their own tale of the pleasures and hardships which are the lot of such enthusiastic sportsmen. The reproductions of Mr. Turner-Turner's original photographs, published by the London Stereoscopic and Photographic Company, Regent Street, are an artistic treat. We are only sorry that no verbal description accompanies them, for this would surely have been most interesting. The photographs, however, faithfully trace the course of the winter spent by Mr. Turner-Turner and his wife in the far North - West. Leaving the Canadian and Pacific Railway at Golden, 300 miles were traversed by steamer, stage and pack horses, far into the heart of the Rocky Mountains, in East Kootenny. Here these intrepid travellers camped for the winter months, building a log hut, the interior presentment of which leads us to conclude that the reply to the second question, "How do you keep warm?" is, "You don't!" and the inside of that log hut after a blizzard is] not to be described in words. Through the whole of the long winter Mr. and Mrs. Turner-Turner saw but one man, the Indian who carried the mail. Two dogs and a cat were their companions through those lonely month. Sports abounded; the deer in such numbers that hundreds might have been killed if needed, and the rivers, the Elk and the Wigwam, running within three yards of the hut, swarming with trout and char, the former, taking the fly throughout the winter whenever the river was free from ice, helped to keep the larder well supplied. The views of river and mountain and forest are superb, and from the picture of "An Idle Day" it is seen that ice and snow were not always paramount. The last photographs show gradually budding leaves, and at last, in May, a pack-train of horses arrives, and a final glimpse of the party is given just starting on their homeward way. The photographs are really beautiful, and the volume is got up to perfection, with its dainty binding and artistic frontispieces. The photographs are too numerous for individual comment, but the reproduction of the one which is here given conveys some idea of the charm of the originals. We should mention that Mr. Turner-Turner is already known as the author of "Three Years' Hunting and Trapping in America and the Great North-West."

BLACK BEAR CAUGHT IN DEADFALL.

INDEX PATHOLOGICUS; FOR THE REGISTRATION OF THE LESIONS RECORDED IN PATHOLOGICAL RECORDS OR CASE BOOKS OF HOSPITALS AND ASYLUMS. By JAMES C. HOWDEN, M.D. (London: J. and A. Churchill.)

The author of this Index, Dr. James C. Howden, deservedly exercises great influence and authority in the district where he resides. Everything he undertakes bears the impress of careful thought and thoroughness. In the "Index Pathologicus" he has supplied an object lesson which will, we hope, be taken to heart by many busy workers in the pathological field, whose duties include the keeping of an index of this kind. Hospital registrars will find this Index suggestive and useful. Its aim is to lessen the labour of research by affording a ready means of reference to the pathological lesions recorded in case books or other records in hospitals and asylums. This object has been well carried out, and the Index will, we hope, find a place in the museum and registrar's department of every well-organised hospital and asylum throughout the country.

HOW TO CORRECT PRINTER'S PROOFS. By the late WILLIAM BLADES. (London: Blades, East, and Blades, Abchurch Lane. 1893.)

All who have "press-proofs" to read have felt troubled from time to time about making the proper marks of correction, "but there is really no difficulty in soon learning all that need be known to make the wishes of the author clear to the printer," Mr. Blades remarks in this handy little volume. And certainly he makes light of all the difficulty of the subject, telling in a very few words what the uninitiated would wish to know. Practical demonstrations are given on the margin of each page of the various technical marks now in vogue, which corrections have indeed been used from the infancy of the art until the present day. This useful little book can be obtained for the sum of 6d., and it is well worth the modest outlay. The correcting of proofs is not a very mysterious art, but even in this a knowledge of technique has its decided advantages.

## Figures and Tables

**Figure f1:**